# Intramuscular and Intradermal Electroporation of HIV-1 PENNVAX-GP^®^ DNA Vaccine and IL-12 Is Safe, Tolerable, Acceptable in Healthy Adults

**DOI:** 10.3390/vaccines8040741

**Published:** 2020-12-07

**Authors:** Srilatha Edupuganti, Stephen C. De Rosa, Marnie Elizaga, Yiwen Lu, Xue Han, Yunda Huang, Edith Swann, Laura Polakowski, Spyros A. Kalams, Michael Keefer, Janine Maenza, Megan C. Wise, Jian Yan, Matthew P. Morrow, Amir S. Khan, Jean D. Boyer, Laurent Humeau, Scott White, Niranjan Y. Sardesai, Mark L. Bagarazzi, Peter B. Gilbert, James G. Kublin, Lawrence Corey, David B. Weiner, on behalf of the HVTN 098 Study Team, the NIAID-Funded HIV Vaccine Trials Network

**Affiliations:** 1 Division of Infectious Disease, Department of Medicine, Emory University, Atlanta, GA 30322, USA; 2 Vaccine and Infectious Disease Division, Fred Hutchinson Cancer Research Center, Seattle, WA 98109, USA; sderosa@fredhutch.org (S.C.D.R.); melizaga@gmail.com (M.E.); ylu2@scharp.org (Y.L.); xhan@scharp.org (X.H.); yunda@scharp.org (Y.H.); jmaenza@fredhutch.org (J.M.); pgilbert@scharp.org (P.B.G.); jkublin@fredhutch.org (J.G.K.); lcorey@fredhutch.org (L.C.); 3 Department of Laboratory Medicine, University of Washington, Seattle, WA 98195, USA; 4 Department of Global Health, University of Washington, Seattle, WA 98195, USA; 5 Division of AIDS, NIH, Bethesda, MD 20892, USA; Swanne@niaid.nih.gov (E.S.); laura.polakowski@nih.gov (L.P.); 6 Vanderbilt University Medical Center, Nashville, TN 37232, USA; s.kalams@vumc.org; 7 Department of Medicine, University of Rochester School of Medicine & Dentistry, Rochester, NY 14642, USA; Michael_Keefer@URMC.Rochester.edu; 8 Division of Allergy and Infectious Diseases, Department of Medicine, University of Washington, Seattle, WA 98195, USA; 9 Inovio Pharmaceuticals Inc. Plymouth Meeting, PA 19462, USA; megan.c.wise@gmail.com (M.C.W.); jyan@inovio.com (J.Y.); matthew.morrow@inovio.com (M.P.M.); amir.khan@merck.com (A.S.K.); jboyer@inovio.com (J.D.B.); lhumeau@inovio.com (L.H.); smwhite64@verizon.net (S.W.); sardesai@geneostx.com (N.Y.S.); mbagarazzi@neuvogen.com (M.L.B.); 10 Wistar Institute, Philadelphia, PA 19104, USA; weinerdavidb@gmail.com

**Keywords:** intramuscular, intradermal, electroporation, HIV vaccine, DNA vaccine, IL-12, safety

## Abstract

*Background:* Several techniques are under investigation to improve the immunogenicity of HIV-1 DNA vaccine candidates. DNA vaccines are advantageous due to their ease of design, expression of multiple antigens, and safety. Methods: The HVTN 098 trial assessed the PENNVAX^®^-GP DNA vaccine (encoding HIV *env*, *gag*, *pol*) administered with or without plasmid IL-12 at 0-, 1-, 3-, and 6-month timepoints via intradermal (ID) or intramuscular (IM) electroporation (EP) in healthy, adult participants. We report on safety, tolerability, and acceptability. Results: HVTN 098 enrolled 94 participants: 85 received PENNVAX^®^-GP and nine received placebo. Visual analog scale (VAS) pain scores immediately after each vaccination were lower in the ID/EP than in the IM/EP group (medians 4.1–4.6 vs. 6–6.5, *p* < 0.01). IM/EP participants reported greater pain and/or tenderness at the injection site. Most ID/EP participants had skin lesions such as scabs/eschars, scars, and pigmentation changes, which resolved within 6 months in 51% of participants (24/55). Eighty-two percent of IM/EP and 92% of ID/EP participant survey responses showed acceptable levels of discomfort. Conclusions: ID/EP and IM/EP are distinct experiences; however, HIV-1 DNA vaccination by either route was safe, tolerable and acceptable by most study participants.

## 1. Introduction

DNA-based immunization is an attractive platform for HIV-1 vaccine development for several reasons [1,2]. First, it has an advantage over live-attenuated vaccine platforms, which may have limitations for use in immunocompromised hosts or people with pre-existing immunity to a vector. Second, the production, scalability and stability of DNA vaccines are generally simpler than other vaccine platforms. Although DNA vaccines are a desirable vaccine modality for HIV-1, they have not elicited sufficient immune responses in humans to advance into efficacy trials, despite their ability to induce adequate humoral and cellular immune responses in preclinical models [1,3]. Several groups have been developing techniques to improve immune responses to DNA vaccines in humans, including codon optimization, the addition of improved leader sequences, highly concentrated DNA formulations, adjuvants, needle-free delivery methods, and electroporation (EP) [4,5]. DNA vaccine delivery via EP can be an efficient way to introduce nucleic acids into cells [6]. The transfection of DNA by in vivo EP exposes the target tissue to a brief electric field pulse that induces temporary and reversible pores in the cell membrane, allowing the entry of macromolecules [7].

In humans, the experimental delivery of vaccines via EP has typically utilized the intramuscular (IM) route involving standard IM injection of plasmid(s) expressing the antigen of interest followed by EP through devices such as CELLECTRA^®^ or Trigrid™ Delivery Systems [8,9,10]. Importantly, an HPV immunotherapeutic DNA vaccine and HIV-1 prophylactic DNA vaccines delivered by IM/EP were safe and well-tolerated, with improved CD4^+^ and CD8^+^ T-cell responses when compared with vaccines without EP [9,10,11]. Newer delivery methods such as intradermal (ID) EP are an exciting platform to pursue given the rich milieu of antigen-presenting cells (dendritic cells, macrophages) in the skin [11]. ID/EP leads to inflammation of the treatment sites and apoptosis or necrosis, which in turn generates an adjuvant effect [12]. Recently, Zika and Ebola DNA vaccines delivered ID/EP by the CELLECTRA^®^-3P device were shown to be safe and immunogenic in heathy human volunteers [13,14].

In addition to EP, the use of cytokine adjuvants have augmented the immunogenicity of DNA vaccines in preclinical studies [15,16,17,18]. Biologically active IL-12 is a pro-inflammatory cytokine, made by macrophages, dendritic cells and B cells, that stimulates the differentiation of naïve CD4 T cells into Th1 cells’ proliferation and polyfunctionality of HIV-specific CD8^+^ T cells in response to antigenic stimulation [16,19,20]. The results of earlier human studies evaluating DNA vaccination with IL-12 DNA (without EP) were safe but did not demonstrate improvement [21]. When given IM/EP, IL-12 DNA administered with HIV PENNVAX^®^-B DNA vaccine in the HIV Vaccine Trials Network (HVTN) 080 study was dose-sparing, with enhanced CD4^+^/CD8^+^ T-cell responses [9].

The primary objective of this study, HVTN 098 (ClinicalTrials.gov NCT02431767), was to determine the safety, tolerability and immunogenicity of the PENNVAX^®^-GP DNA vaccine administered via IM/EP or ID/EP, with or without IL-12 adjuvant. PENNVAX^®^-GP is a combination of plasmids encoding HIV-1 *env, gag, pol*. After an initial pilot safety group of five participants, we assessed safety, tolerability, reactogenicity, acceptability and immune responses in three groups who received PENNVAX^®^-GP: (a) ID/EP without IL-12; (b) ID/EP with IL-12; and (c) IM/EP with IL-12. Immunogenicity findings were described in detail in a separate publication [22]. Here, we report on the safety, tolerability, reactogenicity and acceptability of these regimens in healthy adult volunteers.

## 2. Materials and Methods

### 2.1. Study Participants

**Figure 1:**
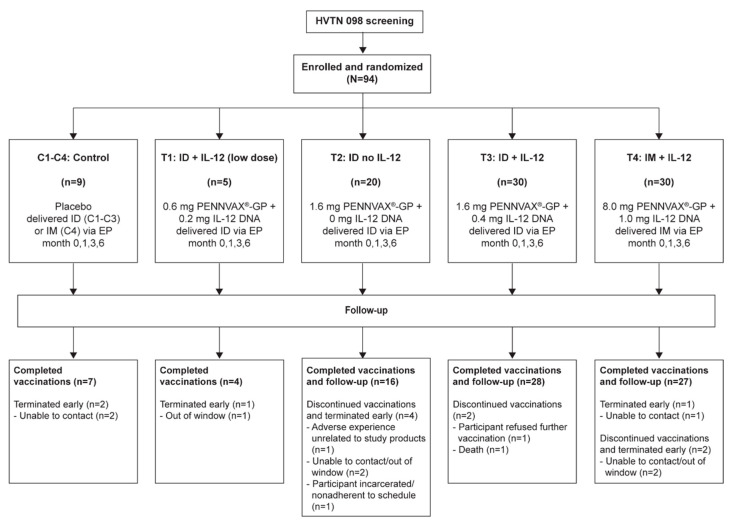
HVTN 098 CONSORT diagram. ID = Intradermal; IM = intramuscular (modified and reproduced with permission from [22].

Participants were healthy, HIV–uninfected (seronegative) adults (age 18–55 years) who provided written informed consent with ongoing informed consent throughout the study. The study was approved and reviewed by the Fred Hutchinson Cancer Research Center Institutional Review Board (IRB), University of Rochester Research Subjects Review Board, Vanderbilt University IRB, and Emory University IRB. Volunteers were recruited, screened, and enrolled if they met eligibility criteria. Enrollment occurred at four US clinical research sites (Atlanta, GA; Nashville, TN; Rochester, NY and Seattle, WA) between September 2015 and June 2016, with completion of follow-up in December 2017. Figure 1 shows a CONSORT diagram of study enrollment and allocation.

### 2.2. Study Agents

The DNA vaccine PENNVAX^®^-GP is a combination of two biologic products with plasmid and codon optimization: SynCon^®^ INO-6112 and SynCon^®^ INO-6145 (see Supplemental Methods). The cytokine adjuvant consisted of a single plasmid, INO-9012 *IL-12* DNA (pIL-12) at 10 mg/mL. PENNVAX^®^-GP and pIL-12 were admixed at appropriate concentrations prior to administration in groups T1, T3 and T4. Placebo was Sterile Water for Injection, USP.

### 2.3. Study Schedule and Dose

The study schedule consisted of four treatment (T1–T4) and control (C1–C4) groups receiving EP injections at four timepoints: months 0, 1, 3 and 6 (Figure 1). Placebo was administered via ID/EP (C1–C3) or IM/EP (C4). T1 was a small pilot safety group of five participants to test the administration of low-dose PENNVAX^®^-GP DNA with pIL-12 via ID/EP at one injection site on the deltoid (0.6 mg PENNVAX-GP, 0.2 mg pIL-12, in 0.1 mL), a cautious approach to evaluate potential skin reactions. Following a safety review of T1 reactogenicity after the first dose, which was uneventful, groups T2, T3 and T4 proceeded with enrollment [23]. T2 received 1.6 mg PENNVAX-GP ID/EP at each vaccination timepoint, divided into two injection sites of 0.1 mL each, and T3 received the same with 0.4 mg pIL-12 in the same volume. T4 received vaccinations via IM/EP at a single injection site (8 mg PENNVAX-GP, 0.2 mg pIL-12 in the skin of deltoid, in 1 mL).

The rationale for T4 was to serve as an IM comparison of the same products given ID/EP in T3, and to expand on the results from HVTN 080, in which participants received 3 mg PENNVAX^®^-B DNA and 1 mg GENEVAX^®^ IL-12 IM/EP [9]. To evaluate if a higher dose of *env* DNA would improve the frequency of Env-specific cellular and humoral responses, a 6-fold higher dose of *env* DNA was used in this study compared with HVTN 080 [9], while doses of *gag* and *pol* plasmids remained the same. Specifically, the T4 PENNVAX^®^-GP dose included 1 mg each of consensus *gag* and *pol* plasmids with 3 mg each of *env* A and *env* C plasmids. A prior clinical trial had tested up to 6 mg DNA IM/EP [24], and up to 8 mg DNA had been given without EP, without safety concerns [23].

An important consideration in choosing the dose of vaccine for ID/EP administration is that ID doses are limited by volume, as 0.1 mL is the maximum ID volume recommended to be given with the EP device at a single injection site, and T3 injections would also need to accommodate the adjuvant volume. At the same time, the dose must be adequate to demonstrate immunogenicity. Several studies of ID vs IM administration of other vaccines (without EP) had used one-tenth to one-fifth of the IM dose to be given as the ID dose, and found those to be similarly safe and immunogenic to the full dose IM [25,26,27]. The concentration of each DNA product in this study was 10 mg/mL. The ID/EP dose of PENNVAX^®^-B DNA was 1/5 the IM/EP dose, which was as much as could be accommodated within a volume of 0.2 mL divided into two injection sites. As in the IM/EP group, the PENNVAX-GP doses given ID/EP were similarly weighted towards *env*, containing 0.2 mg each consensus *gag* and *pol* plasmids and 0.6 mg each of env A and *env C* plasmids.

### 2.4. Electroporation

The CELLECTRA^®^ adaptive constant current device is a portable, battery-powered device designed to facilitate the introduction of DNA into the muscle or skin via EP. The CELLECTRA^®^ 3P EP system was used for ID delivery, and the CELLECTRA^®^ 5P EP system was used for IM delivery (see Supplemental Methods). The CELLECTRA^®^ pulse generator is tethered to an applicator with disposable IM or ID arrays [28]. Additional details on the EP devices were reported by Diehl et al. [11].

### 2.5. Safety and Tolerability Assessments

Safety was assessed by solicited local and systemic reactogenicity signs and symptoms at 25–60 min post-injections, followed by participant self-report daily for seven days. Solicited symptoms included: injection site pain, tenderness, erythema, induration or swelling, malaise and/or fatigue, myalgia, headache, chills, arthralgia, nausea, and vomiting. The deltoid injection sites were inspected at each scheduled visit and photographed. Skin lesions were enumerated, measured and recorded using the following terms: papule, blister, macule, flat scar, scab/eschar, raised scar, keloid, hypopigmentation, hyperpigmentation, or ‘other.’ Adverse events (AEs) were reported for 12 months after first vaccine. Additional contacts were made at months 24 and 36 months after first vaccination for solicited information on serious AEs (SAEs), other important medical events. Reactogenicity symptoms and AEs were scored using the DAIDS Table for Grading the Severity of Adult and Pediatric Adverse Events (version 2.0, November 2014, National Institutes of Health, Bethesda, MD, USA). Routine clinical laboratory tests included: complete blood count with differential and platelets, alanine aminotransferase, aspartate aminotransferase (AST), alkaline phosphatase, creatinine, creatine kinase, and urinalysis.

Magnitude of pain with EP was assessed at 0, 5–7, and 25–60 min after each vaccination, using a visual analog scale (VAS). Participants rated their injection site pain on a 10-point scale (the pain score) using a 10-cm line, with one end marked “no pain” and the other end marked “worst pain.” VAS is the most commonly used and validated scale to measure self-reported pain in clinical and surgical patients [29], demonstrating high reproducibility and sensitivity [30].

As a measure of EP acceptability, two weeks following each injection and at six months after the fourth vaccination, participants completed a questionnaire that asked questions regarding acceptability and their willingness to undergo EP.

### 2.6. Statistical Methods

Sample size was determined to provide reasonable precision in the assessment of the primary safety and immunogenicity endpoints. Group T1 was enrolled first. The randomization allocation sequence for groups T2–T4, done in blocks to ensure balance across arms, was obtained by computer-generated random numbers and provided to each clinical site through a web-based randomization system. At each site, the pharmacist with primary responsibility for dispensing study products was charged with maintaining security of the treatment assignments within each group. Participants and site staff (except for site pharmacists) were blinded as to treatment arm assignments (vaccine vs. placebo) within each group, but unblinded to participant group assignments. All randomized participants were included in the safety analyses.

Barnard’s exact and Wilcoxon rank sum tests were used to compare the related adverse event rates and VAS scores, respectively, between two groups. The False Discovery Rate (FDR) adjustment [31] was used to correct for multiple comparisons of related adverse events and VAS scores between two groups. All figures were created in R version 3.5.3, and all statistical tests were performed in SAS Version 9.4 (Cary, CA, USA). A *p*-value ≤ 0.05 is considered statistically significant.

## 3. Results

### 3.1. Study Population Characteristics

HVTN 098 enrolled 94 participants; nine received placebo (six via ID/EP (C1-C3) and three via IM/EP (C4)) and 85 received vaccine (Figure 1). The median age was 28 years (range 18–54) and 56% were assigned male sex at birth (Table 1). Participant population was 9% black/African American, 3% Asian, 7% multiracial, and 5% Hispanic/Latinx.

Of the 94 participants, 85 (90%) received all scheduled vaccinations. There was >90% adherence among all treatment and placebo groups, except only 80% of participants received vaccination #3 in T1 (4/5) and vaccination #4 in T2 (16/20) (Table 1). Seven participants discontinued vaccinations and terminated the study early (Figure 1).

### 3.2. Pain Scores and Reactogenicity

#### 3.2.1. VAS Scores

**Figure 2:**
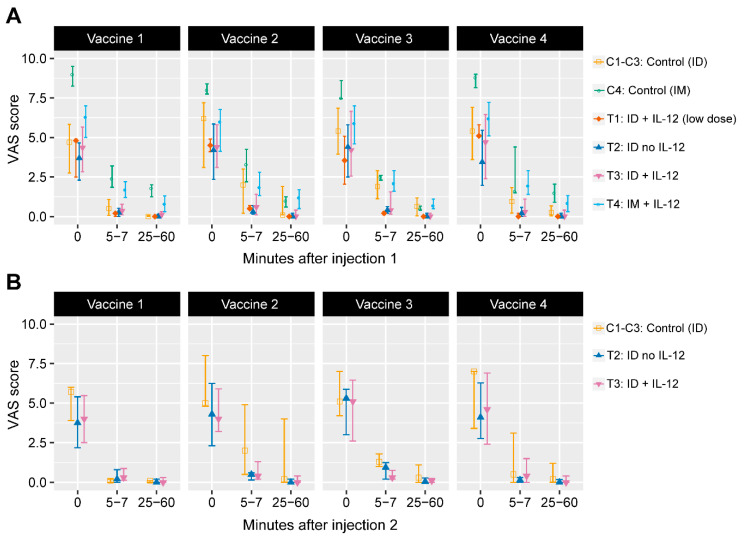
Distributions (median, 25th and 75th percentiles) of visual analog scale (VAS) scores by treatment group. VAS scores immediately following injection at time 0 min, 5 to 7 min later, and 25 to 60 min later at (**A**) injection 1 site, and (**B**) injection 2 site on the opposite deltoid. ID = Intradermal; IM = intramuscular. C1–C4 served as the control groups for each of the four treatment groups T1–T4.

VAS pain scores immediately following each injection (0 min), 5–7 min later and 25–60 min later are shown in Figure 2A (after injection 1) and Figure 2B (after injection 2). Injections 1 and 2 were given within 5 min of each other. VAS scores were significantly lower in the ID groups (median 4.1–4.6) than in the IM group (median 6–6.5) immediately after each vaccination (all adjusted *p*-values <0.01, Supplementary Table S1). Five to seven and 25–60 min after vaccination, VAS scores precipitously decreased in all groups as expected, but remained higher in the IM/EP group compared with the ID/EP groups. At 25–60 min after vaccination, ID/EP participants had no or minimal pain scores (median of zero), and IM/EP participants reported pain scores with a median of 0.7–1.2. Participants in T2 and T3 received a second ID/EP injection in the opposite deltoid for completion of antigen delivery. Median VAS scores immediately following the second injection were similar to the first (Figure 2B). Participants receiving placebo, regardless of ID or IM injection, had a wide interquartile range of VAS scores, likely due to small sample size (Figure 2).

#### 3.2.2. Reactogenicity

**Figure 3:**
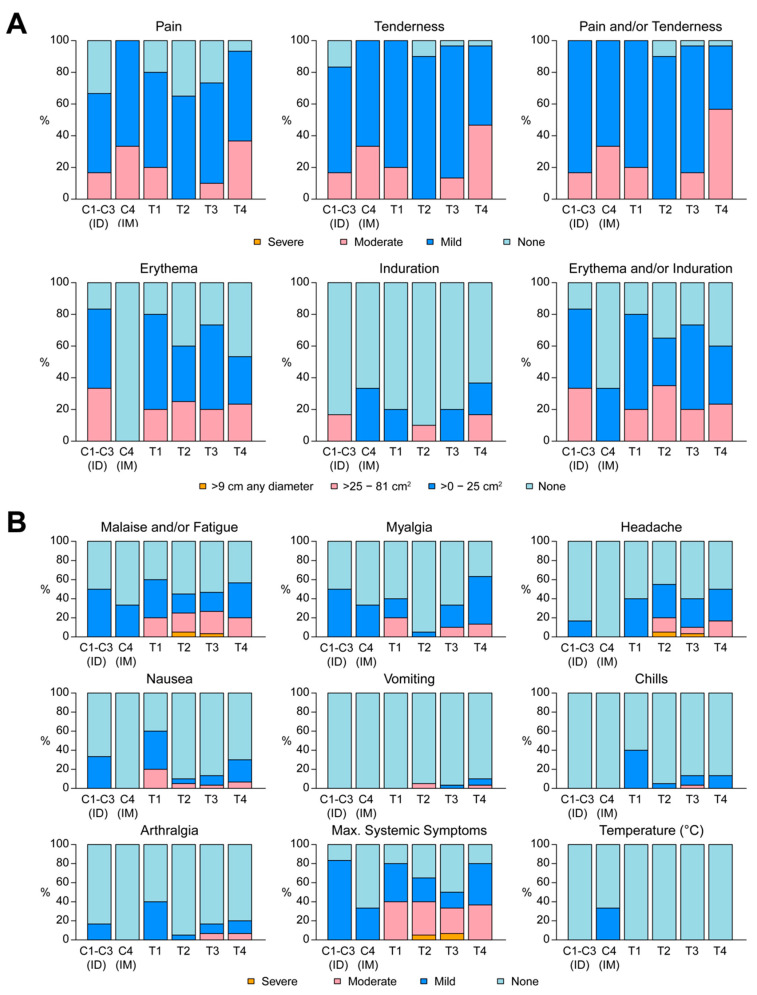
Local and systemic reactogenicity. (**A**) Maximum solicited local reactogenicity (pain, tenderness, erythema, induration) by treatment group. Bar graphs show the percentage of participants in each group reporting the specified maximum severity during the reactogenicity period (7 days). (**B**) Maximum solicited systemic reactogenicity (malaise/fatigue, myalgia, headache, nausea, vomiting, chills, arthralgia) by treatment group. ID = Intradermal; IM = intramuscular. C1–C4 served as the control groups for each of the four treatment groups T1–T4.

Local injection site reactogenicity assessments were similar across the groups, except for pain and tenderness, which were significantly greater in T4 (*p* < 0.01) (Figure 3A). In T3 (ID/EP) 3/30 participants vs. 11/30 in T4 (IM/EP) reported moderate/severe pain. Similarly, 14/30 participants in T4 vs. 4/30 in T3 had moderate/severe tenderness. Injection site erythema and induration were similar across the groups (*p* = 0.34) (Figure 3A).

The degree of maximum systemic reactogenicity symptoms was not significantly different across treatment groups (Figure 3B). Most participants experienced mild or moderate systemic symptoms, except for three: one participant in T2 experienced severe malaise and/or fatigue and headache, one participant in T3 experienced a severe headache, and another experienced severe malaise and/or fatigue. There were no life-threating reactogenicity events.

**Figure 4:**
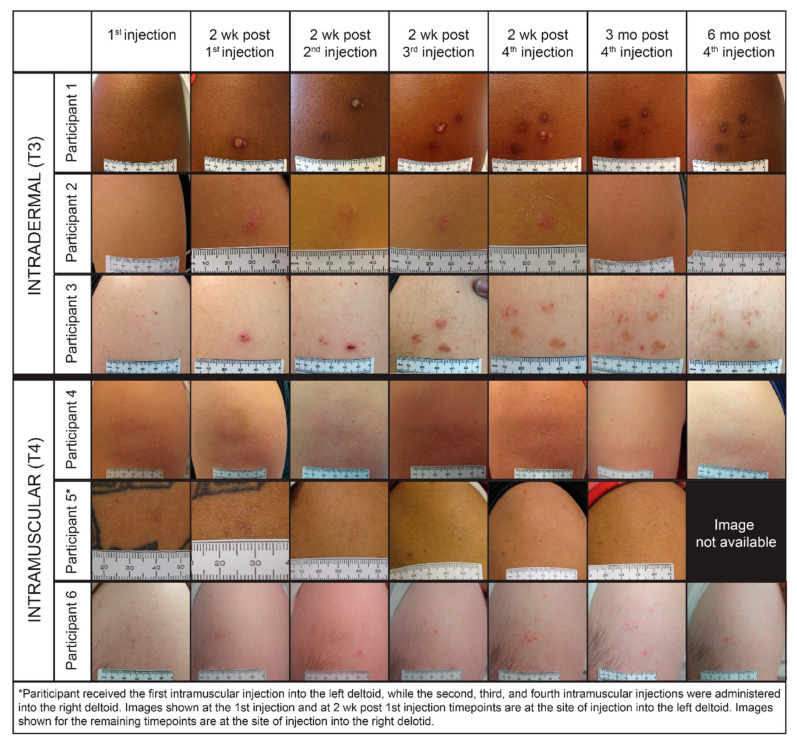
Injection site-related skin changes. Injection site-related skin changes shown in three representative participants from each of the intradermal and intramuscular groups. Wk = week; mo = month.

Injection site lesions were visible at the two weeks post-vaccination timepoint in 100% of ID/EP participants including ID/EP controls. Scab/eschar and flat scar were the most common lesions observed (Supplementary Table S2). At six months after the last ID/EP injection, 49% of participants had skin lesions (Figure 4). Scab/eschar development was more common in T3 (ID/IL-12) compared with ID alone in T2 (56 vs. 21 lesions, *p* < 0.05). In T4, injection site skin lesions were minimal except for bruising and pruritus (Figure 4). Injection site skin lesions were highly variable and markedly different among individuals regardless of skin tone (Figure 4). Participants with history of hypertrophic scars or keloids were excluded from enrolling in this study.

### 3.3. Adverse Events

Thirty-four participants experienced at least one AE that was reported as related to the vaccine, including injection site pruritis, bruising, discharge, presyncope, procedural anxiety, lymphadenopathy, muscle weakness, and increased AST (Supplementary Table S3). One person in T3 discontinued vaccinations due to pain, anxiety, and a presyncopal episode after ID/EP. Injection site bruising was more common in the IM/EP than ID/EP groups. Placebo recipients (*n* = 9) did not report any AEs related to study product. There were no SAEs related to study product. One death occurred (T3) related to opiate and alcohol overdose.

### 3.4. Acceptability of Electroporation

Acceptability questionnaires were collected at five serial timepoints during the study and were pooled for analysis: 82% of responses from IM/EP and 92% from ID/EP groups noted similar levels of anxiety and the level of discomfort was acceptable (Table 2). Overall, 99% and 93% of ID/EP and IM/EP participants’ responses, respectively, stated they would recommend EP vaccination of an effective HIV vaccine to their family/friends if it felt the same as their trial experience (Table 3. However, fewer than 50% of IM/EP participants’ responses indicated that they would recommend EP if it were more painful than their experience, if the recipients were children <16 years of age, if the vaccine was only 30% effective, or for people at low risk of HIV-1 infection (Table 3)), whereas at least 61% of ID/EP participants’ responses indicated that they would continue to recommend EP under each of these circumstances. Post-vaccination injection site skin changes were also found to be acceptable and did not lead to discontinuation of the vaccine regimen.

We asked participants whether they would be willing to undergo EP (1) if it were required for a new vaccine against a serious disease (if they were at risk for that disease), and (2) if it increased the effectiveness of an existing vaccine such as influenza. For question 1, all except for one ID/EP participant and one IM/EP participant said they were willing to undergo EP. These two participants said they were “probably not willing” to undergo EP. For question 2, 14/61 (23%) participants in the ID/EP group and 12/33 (36%) participants in the IM/EP group stated they were either “probably not willing” or “definitely not willing” to undergo EP.

The HVTN routinely gathers information on the social impact of participation in HIV vaccine studies. Most of the participants (90/94) reported beneficial impacts such as feeling good helping others, compensation and increased awareness and knowledge about research. Four participants reported negative social impacts of participation affecting personal relationships, which were rated as mild. All were resolved, except for one participant whose parent was unsupportive of trial participation.

## 4. Discussion

HVTN 098 is the first study to compare administration of the PENNVAX^®^-GP DNA vaccine via ID/EP and IM/EP. We have demonstrated that both approaches are safe, tolerable, and acceptable among healthy adults. We have also shown that administration of the pIL-12 adjuvant via ID/EP is safe and tolerable. There were no serious safety events related to the vaccines or mode of administration. This is consistent with the excellent safety profile observed in several other phase 1 studies of IM/EP DNA vaccines for infectious diseases [9,10,13,14,24,32,33].

At the time of this study, there had been limited previous experience with ID/EP delivery of pIL-12 and potential side effects. Investigational intratumor pIL-12 ID/EP injections had been given to melanoma patients, in which necrosis at the injection sites was a desired effect. In healthy volunteers, the potential for injection site necrosis was a concern [34]. Fortunately, serious injection site reactions did not arise. In our study, injection site lesions were appreciably more frequent in the ID/EP groups compared with IM/EP, and were also seen in ID/EP placebo recipients and generally faded over time. Two participants in the ID/EP group had a small amount of purulent discharge at the injection site—also noted in an ID/EP participant in the Zika vaccine trial—likely due to an inflammatory reaction beneath an eschar [13]. Skin changes persisted in some ID/EP volunteers six months after the last vaccination. However, no injection site changes were seen that were a clinical concern; no one discontinued vaccinations due to cosmetic effects, and no one reported social impacts related to the appearance of injection sites. The IM/EP group experienced minimal injection site changes at any timepoint, and no persistent lesions.

Despite the more prominent injection side effects in the ID/EP groups, 96% of responses on acceptability questionnaires said the appearance of the sites was acceptable, 98% indicated they would recommend EP for an effective HIV vaccine even if it left a transient scar, and 94% indicated they would recommend even if it left a permanent scar (compared with 85% of responses from the IM/EP group). One limitation of our study is that we did not include IM/EP without the pIL-12 group, instead we referred to an equivalent group in HVTN 080 study which used a slightly different DNA construct in the vaccine.

Overall, vaccinations were well-tolerated among all groups. EP is painful at the time of administration but discomfort resolves within minutes. As expected, participants in the IM/EP group had higher VAS scores for pain when compared with ID/EP, with a median of 6–6.5 for IM/EP and 4.1–4.6 for ID/EP. VAS scores in HVTN 080 with the CELLECTRA^®^SP device and PENNVAX^®^-B DNA vaccine were a median of 5–5.4 for IM/EP. The increased VAS scores in this study could be due to the higher dose of DNA (8 vs. 3 mg of PENNVAX), or differences in formulation, as PENNVAX^®^-B was formulated in a citrate buffer including 0.25% bupivacaine-HCl whereas PENNVAX^®^-GP is diluted in sterile water [10]. Fifty percent of participants in the IM/EP group in this study reported moderate to severe pain and tenderness, which was similar to the findings in HVTN 080 and RV262, both of which used the CELLECTRA^®^ device and similar PENNVAX^®^ DNA vaccines [9,33]. In prior vaccine trials, HVTN 080 and the HPV study using CELLECTRA^®^ IM/EP, a few participants also discontinued due to pain and tenderness [9,24]. In HVTN 087, which used a TriGrid Delivery System (TDS) EP device, lower pain scores were noted, possibly because the vaccine formulation contained bupivacaine to enhance DNA uptake and expression [10]. No local pain relief measures were used in our study. TDS-IM was also used to assess a different HIV-1 DNA vaccine with/without IL-12 DNA in an African population; 46.7% (35/75) reported pain as “uncomfortable” and 12% (9/75) reported pain as “intense” immediately following vaccination [35]. Overall, IM/EP is more painful than ID/EP at the time of administration, likely due to a large muscle contraction (such as the deltoid), but the pain diminishes rapidly after 5 min.

## 5. Conclusions

HIV-1 DNA vaccines delivered via IM/EP or ID/EP are safe, tolerable and acceptable. ID/EP is dose-sparing, using 1/5 of the IM/EP dose, and is a promising approach to vaccine development. In addition, CD4 and CD8 T cell responses were equivalent in ID/IL-12 vs IM/IL-12 groups and the magnitude of antibody responses to consensus gp140 was higher in the ID/IL-12 group [22]. ID/EP, while less painful than IM/EP, leads to various skin lesions at the injection site that may still be seen six months post-last vaccination. However, these effects were found to be acceptable by our study population. Acceptability and willingness to undergo EP appear to be influenced by the seriousness or importance of the disease and the availability of a vaccine for that disease. Participants in preventative HIV-1 vaccine trials are motivated by a variety of complex factors including altruism [36]. Further exploration of EP delivery of DNA vaccines in the context of new and emerging serious infections such as COVID-19 and their acceptability among the general population warrants further exploration.

## Figures and Tables

**Table 1:** HVTN 098 study population demographics and vaccination frequencies.

Category	Control: C1-C4 (n = 9)	T1: ID low dose (n = 5)	T2: ID no IL-12 (n = 20)	T3: ID + IL-12 (n = 30)	T4: IM + IL-12 (n = 30)	Total (n = 94)
Sex						
Male	4 (44%)	4 (80%)	12 (60%)	20 (67%)	13 (43%)	53 (56%)
Female	5 (56%)	1 (20%)	8 (40%)	10 (33%)	17 (57%)	41 (44%)
Race						
White	7 (78%)	5 (100%)	19 (95%)	24 (80%)	21 (70%)	76 (81%)
Black/African American	1 (11%)	0 (0%)	0 (0%)	4 (13%)	3 (10%)	8 (9%)
Asian	1 (11%)	0 (0%)	1 (5%)	0 (0%)	1 (3%)	3 (3%)
Native Hawaiian/Pacific Islander	0 (0%)	0 (0%)	0 (0%)	0 (0%)	0 (0%)	0 (0%)
Native American/Alaskan Native	0 (0%)	0 (0%)	0 (0%)	0 (0%)	0 (0%)	0 (0%)
Multiracial	0 (0%)	0 (0%)	0 (0%)	2 (7%)	5 (17%)	7 (7%)
Other	0 (0%)	0 (0%)	0 (0%)	0 (0%)	0 (0%)	0 (0%)
Ethnicity						
Hispanic or Latino/a	1 (11%)	0 (0%)	0 (0%)	1 (3%)	3 (10%)	5 (5%)
Non-Hispanic or Latino/a	8 (89%)	5 (100%)	20 (100%)	29 (97%)	27 (90%)	89 (95%)
Age (Years)						
Less than 18	0 (0%)	0 (0%)	0 (0%)	0 (0%)	0 (0%)	0 (0%)
18–20	0 (0%)	0 (0%)	2 (10%)	3 (10%)	3 (10%)	8 (9%)
21–30	6 (67%)	3 (60%)	9 (45%)	13 (43%)	17 (57%)	48 (51%)
31–40	2 (22%)	1 (20%)	7 (35%)	5 (17%)	2 (7%)	17 (18%)
41–50	1 (11%)	0 (0%)	2 (10%)	8 (27%)	7 (23%)	18 (19%)
Over 50	0 (0%)	1 (20%)	0 (0%)	1 (3%)	1 (3%)	3 (3%)
Median	27.0	30.0	27.0	30.0	28.0	28.0
Range	23–45	24–54	18–45	18–51	18–54	18–54
Vaccination Frequencies						
Day 0 (Vaccine 1)	9 (100%)	5 (100%)	20 (100%)	30 (100%)	30 (100%)	94 (100%)
Day 28 (Vaccine 2)	9 (100%)	5 (100%)	19 (95%)	29 (97%)	30 (100%)	92 (98%)
Day 84 (Vaccine 3)	9 (100%)	4 (80%)	18 (90%)	27 (90%)	29 (97%)	87 (93%)
Day 168 (Vaccine 4)	9 (100%)	5 (100%)	16 (80%)	27 (90%)	28 (93%)	85 (90%)

Abbreviations: ID = Intradermal; IM = intramuscular. C1–C4 served as the control groups for each of the four treatment groups T1–T4.

**Table 2:** Acceptability of intramuscular (IM) and intradermal (ID) vaccination with electroporation (EP). Responses from two weeks after each injection and six months after final injection, combined.

Question	Acceptable	Unacceptable	Don’t Know/Don’t Remember
Level of discomfort during vaccination with EP	ID 262/286.92% IM 127/155.82%	ID 22/286.8% IM 22/155.14%	ID 2/286.1% IM 6/155.4%
Level of discomfort during the week after vaccination with EP	ID 285/286.100% IM 154/155.99%	ID 1/286.0% IM 1/155.1%	ID 0/286.0% IM 0/155.0%
Level of anxiety with EP vaccination	ID 271/286.95% IM 135/155.87%	ID 14/286.5% IM 10/155.6%	ID 1/286.0% IM 10/155.6%
Appearance of the injection site	ID 274/286.96% IM 152/155.98%	ID 10/286.3% IM 2/155.1%	ID 2/286.1% IM 1/155.1%
Any other side effects experienced from vaccination with EP? ^a^	ID 42/286.15% IM 19/155.12%	ID 1/286.0% IM 1/155.1%	ID 1/286.0% IM 3/155.2%

^a^ No other side effects: ID 242/286, 85%; IM 132/155, 85%. Abbreviations: EP = electroporation; ID = Intradermal; IM = intramuscular.

**Table 3:** Participant willingness to recommend electroporation (EP) vaccination to others, for an effective HIV vaccine, under specific circumstances. Responses from two weeks after each injection and six months after final injection, combined.

Question	Yes	No	Don’t Know
Would you recommend in the real world if it would feel exactly the same as your experience?	ID 283/286.99% IM 144/155.93%	ID 0/286.0% IM 3/155.2%	ID 3/286.1% IM 8/155.5%
Would you recommend in the real world if it would feel more painful than your experience?	ID 203/286.71% IM 77/155.50%	ID 31/286.11% IM 32/155.21%	ID 52/286.18% IM 46/155.30%
Would you recommend in the real world if it would leave a mark on the skin lasting for weeks?	ID 280/286.98% IM 149/155.96%	ID 3/286.1% IM 3/155.2%	ID 3/286.1% IM 3/155.2%
Would you recommend in the real world if it would leave a permanent scar?	ID 269/286.94% IM 134/155.86%	ID 2/286.1% IM 3/155.2%	ID 15/286.5% IM 18/155.12%
Would you recommend in the real world if the vaccine would be 50% effective?	ID 225/286.79% IM 100/155.65%	ID 34/286.12% IM 9/155.6%	ID 27/286.9% IM 46/155.30%
Would you recommend in the real world if the vaccine would be 30% effective?	ID 175/286.61% IM 43/155.28%	ID 67/286.23% IM 48/155.31%	ID 44/286.15% IM 64/155.41%
Would you recommend in the real world if the person receiving the vaccine is under 16?	ID 213/286.74% IM 68/155.44%	ID 30/286.10% IM 40/155.26%	ID 43/286.15% IM 47/155.30%
Would you recommend in the real world if the person is your family member or friend?	ID 280/286.98% IM 147/155.95%	ID 1/286.0% IM 2/155.1%	ID 5/286.2% IM 6/155.4%
Would you recommend the vaccine if the person is at low risk for infection?	ID 175/286.61% IM 54/155.35%	ID 62/286.22% IM 52/155.34%	ID 49/286.17% IM 49/155.32%

Abbreviations: EP = electroporation; ID = Intradermal; IM = intramuscular.

## Supplementary Materials

The following are available online at https://www.mdpi.com/2076-393X/8/4/741/s1, Table S1: Summary of pain scores following each vaccination, assessed by visual analog scale (VAS), Table S2: Injection site skin changes in the Intradermal and Intramuscular groups [total number of lesions at the electroporation (EP) injection site in the treatment and control groups], Table S3: Related adverse events (AE) in treatment groups by route of administration.
