# Supplementary material for: Intramuscular and Intradermal Electroporation of HIV-1 PENNVAX-GP® DNA Vaccine and IL-12 Is Safe, Tolerable, Acceptable in Healthy Adults
Source: Vaccines (Basel). 2020 Dec 7;8(4):741. doi: 10.3390/vaccines8040741 (PMC7762306; doi:10.3390/vaccines8040741)
Supplement: Supplementary file 1 [file vaccines-08-00741-s001.pdf]

vaccines

MDPI

Supplementary Materials

Members of the HVTN 098 study team:

Colleen Kelley [1], Mark Mulligan [1], Nadine Rouphael [1], Kyle Rybczyk [2], Amanda Langlois [2], Shonda Sumner [2], M. Juliana McElrath [3], David Berger [3], Cynthia Monaco [4], and Catherine Bunce [4]

[1] Division of Infectious Disease, Department of Medicine, Emory University, Atlanta, Georgia, United States of America
[2] Vanderbilt University Medical Center, Nashville, Tennessee, United States of America
[3] Vaccine and Infectious Disease Division, Fred Hutchinson Cancer Research Center, Seattle, Washington, United States of America
[4] Department of Medicine, University of Rochester School of Medicine & Dentistry, Rochester, New York, United States of America

Table S1. Summary of pain scores following each vaccination, assessed by visual analog scale (VAS).

Vaccine #	Minutes after vaccination	VAS score ID/EP n	VAS score ID/EP Median (range)	VAS score IM/EP n	VAS score IM/EP Median (range)	p-value[a] (ID vs. IM)	FDR-adjusted q-value
1	0	61	4.1 (0.4, 10)	32	6.55 (2.0, 10)	<0.001	<0.001
	5-7		0.3 (0, 1.6)		1.75 (0.3, 4.4)	<0.001	<0.001
	25-60		0 (0, 2.2)		0.8 (0, 2.7)	<0.001	<0.001
2	0	58	4.4 (0, 9.5)	33	6.0 (2.1, 10)	0.007	0.007
	5-7		0.5 (0, 4.8)		1.9 (0.4, 6.3)	<0.001	<0.001
	25-60		0 (0, 2)		1.2 (0, 4.6)	<0.001	<0.001
3	0	55	4.4 (0.2, 9)	32	6.1 (1.1, 10)	0.005	0.006
	5-7		0.4 (0, 5.7)		2.1 (0.2, 6.0)	<0.001	<0.001
	25-60		0 (0, 2)		0.7 (0.1, 3.5)	<0.001	<0.001
4	0	54	4.6 (0.3, 9.9)	31	6.5 (1.2, 10)	0.003	0.003
	5-7		0.3 (0, 5.8)		1.9 (0.4, 7.2)	<0.001	<0.001
	25-60		0 (0, 2.8)		0.9 (0.1, 4.2)	<0.001	<0.001

[b]p-values are calculated based on Wilcoxon rank-sum test. Abbreviations: VAS = visual analog scale; EP = electroporation; ID = Intradermal; IM = intramuscular; FDR = False Discovery Rate.

Table S2. Injection site skin changes in the Intradermal and Intramuscular groups [total number of lesions at the electroporation (EP) injection site in the treatment and control groups].

	ID/EP (T1+T2+T3, n=55)			ID/EP (Control: C1-C3, n=6)			IM/EP (T4, n=30)			IM/EP (Control: C4, n=3)		
	2 wk post 1st inj	3 mo post 4th inj	6 mo post 4th inj	2 wk post 1st inj	3 mo post 4th inj	6 mo post 4th inj	2 wk post 1st inj	3 mo post 4th inj	6 mo post 4th inj	2 wk post 1st inj	3 mo post 4th inj	6 mo post 4th inj
**No. of participants withskin lesions/total no. assessed**	55/55	32/55	26/55	6/6	4/6	4/6	16/30	5/30	2/30	1/3	0/3	0/3
**Papule**	1	0	0	0	0	0	0	0	0	0	0	0
**Blister/Vesicle**	0	0	0	0	0	0	0	0	0	0	0	0
**Macule**	2	0	0	0	0	0	8	0	0	0	0	0
**Flat scar**	51	112	158	1	0	0	6	5	0	0	0	0
**Scab/Eschar**	85	0	0	20	0	0	17	0	0	3	0	0
**Raised scar**	24	3	5	3	8	18	0	0	0	0	0	0
**Keloid**	0	0	0	0	0	0	0	0	0	0	0	0
**Hypopigmentation**	0	7	11	0	17	27	5	10	4	0	0	0
**Hyperpigmentation**	7	47	26	0	28	5	4	4	1	0	0	0
**Other**	28	22	17	4	3	8	14	3	0	0	0	0

Abbreviations: EP = electroporation; ID = Intradermal; IM = intramuscular; wk = week; mo = month.

Table S3. Related adverse events (AE) in treatment groups by route of administration[a].

AE term	ID/EP (T1+T2+T3, n=55)	IM/EP (T4) (T4, n=30)	Combined (n=85)	Raw P-value**	FDR Adjusted P-value
Participants with one or more AEs	18 (32.7%)	16 (53.3%)	34 (36.2%)	0.072	0.359
Injection site pruritus	10 (18.2%)	8 (26.7%)	18 (19.1%)	0.415	0.577
Injection site bruising	0 (0.0%)	6 (20.0%)	6 (6.4%)	<0.001	0.005
Presyncope	4 (7.3%)	1 (3.3%)	5 (5.3%)	0.577	0.577
Lymphadenopathy	1 (1.8%)	2 (6.7%)	3 (3.2%)	0.318	0.577
Injection site discharge	2 (3.6%)	0 (0.0%)	2 (2.1%)	0.404	0.577
Aspartate aminotransferase increased	1 (1.8%)	0 (0.0%)	1 (1.1%)	0.568	0.577
Muscular weakness	1 (1.8%)	0 (0.0%)	1 (1.1%)	0.568	0.577
Procedural anxiety	0 (0.0%)	1 (3.3%)	1 (1.1%)	0.188	0.470
Pruritus	0 (0.0%)	1 (3.3%)	1 (1.1%)	0.188	0.470

[a]Participants in control groups (n=9) did not have adverse events related to study product. [b]Raw p-values are calculated based on Barnard's test. Abbreviations: AE = adverse event; EP = electroporation; ID = Intradermal; IM = intramuscular; FDR = False Discovery Rate.

Supplemental Methods

Study agents

The DNA vaccine PENNVAX®-GP is a combination of two biologic products: SynCon® INO-6112 (consisting of two plasmids encoding HIV-1 clade A consensus *env* [pGX1001] and clade C consensus *env* [pGX1002]) and SynCon® INO-6145 (consisting of two plasmids encoding multi-clade consensus *pol* [pGX1004] and *gag* [pGX1005]). All plasmids were manufactured by VGXI (The Woodlands, TX).

The cytokine adjuvant consisted of a single plasmid, pGX6001 (INO-9012), containing a dual promoter system for expression of both the human IL-12 p35 and p40 subunits necessary for production of the active heterodimeric IL-12 protein. The p35 subunit was under the control of the hCMV promoter/enhancer and SV40 polyadenylation signal, whereas the p40 subunit was under the control of the simian CMV promoter and BGH polyadenylation signal. INO-9012 *IL-12* DNA (pIL-12) was at a concentration of 10 mg/mL and manufactured by VGXI (The Woodlands, Texas). PENNVAX®-GP and pIL-12 were admixed at the appropriate concentrations prior to administration in groups T1, T3 and T4.

The placebo was Sterile Water for Injection, USP. As the DNA vaccine was diluted in sterile water, sterile water was chosen as the placebo to minimize the possibility of unintended unblinding, and to more closely match the study product.

Electroporation

For IM/EP, the operator inserted the applicator onto the surface of the subject's arm, performed a 1 mL volume IM injection into the deltoid muscle through a portal in the center of the array, and pushed a trigger to initiate the EP procedure (3 pulses, 0.5 Amp, 52 ms, 1 sec between pulses). The electric field was generated within the area surrounding the IM injection through five 21-gauge 19 mm needle electrodes in a 1 cm diameter pentagonal shape. For ID/EP, the applicator had a disposable array with three 26-gauge 3 mm needle electrodes. The operator injected 0.1 mL of the vaccine ID into the skin overlying the deltoid muscle (using a 25-gauge needle with a length of 5/8 inches) creating a bleb/wheal, followed by insertion of the ID applicator directly onto the bleb. The trigger was then activated to initiate the EP procedure (4 pulses, 0.2 Amps, 52 ms, 0.2 sec between pulses).

Supplemental Results

Administration errors with EP devices

Administration errors and technical difficulties of IM/EP and ID/EP DNA delivery were tracked since the procedure requires training (349 total injections). IM/EP resulted in more administration errors (six errors) than ID/EP (one error). IM/EP involved a needle and syringe administration of vaccine through a channel in the applicator and then pressing the trigger for EP, which could be cumbersome. As such, most errors occurred when the EP was inadvertently given before the product was injected. Of note, almost all the errors occurred with vaccine #1 or #2, potentially due to inexperience of the operator performing the procedure in the early period of the trial. No AEs were reported attributed to vaccine administration errors.
